# Rapid detection and subtyping of European swine influenza viruses in porcine clinical samples by haemagglutinin‐ and neuraminidase‐specific tetra‐ and triplex real‐time RT‐PCRs

**DOI:** 10.1111/irv.12407

**Published:** 2016-08-09

**Authors:** Dinah Henritzi, Na Zhao, Elke Starick, Gaelle Simon, Jesper S. Krog, Lars Erik Larsen, Scott M. Reid, Ian H. Brown, Chiara Chiapponi, Emanuela Foni, Silke Wacheck, Peter Schmid, Martin Beer, Bernd Hoffmann, Timm C. Harder

**Affiliations:** ^1^ Institute of Diagnostic VirologyFriedrich‐Loeffler‐Institute (FLI)Greifswald‐Insel RiemsGermany; ^2^ AnsesPloufragan‐Plouzané LaboratorySwine Virology Immunology UnitPloufraganFrance; ^3^ National Veterinary Institute; Technical University of Denmark (DTU)Frederiksberg CDenmark; ^4^ Department of VirologyAnimal and Plant Health Agency‐WeybridgeNew HawAddlestoneSurreyUK; ^5^ Istituto Zooprofilattico Sperimentale della Lombardia ed Emilia RomagnaParmaItaly; ^6^ IDT Biologika GmbHDessau‐RosslauGermany

**Keywords:** influenza A virus, multiplex RT‐qPCR, subtyping, surveillance, swine, zoonosis

## Abstract

**Background:**

A diversifying pool of mammalian‐adapted influenza A viruses (IAV) with largely unknown zoonotic potential is maintained in domestic swine populations worldwide. The most recent human influenza pandemic in 2009 was caused by a virus with genes originating from IAV isolated from swine. Swine influenza viruses (SIV) are widespread in European domestic pig populations and evolve dynamically. Knowledge regarding occurrence, spread and evolution of potentially zoonotic SIV in Europe is poorly understood.

**Objectives:**

Efficient SIV surveillance programmes depend on sensitive and specific diagnostic methods which allow for cost‐effective large‐scale analysis.

**Methods:**

New SIV haemagglutinin (HA) and neuraminidase (NA) subtype‐ and lineage‐specific multiplex real‐time RT‐PCRs (RT‐qPCR) have been developed and validated with reference virus isolates and clinical samples.

**Results:**

A diagnostic algorithm is proposed for the combined detection in clinical samples and subtyping of SIV strains currently circulating in Europe that is based on a generic, M‐gene‐specific influenza A virus RT‐qPCR. In a second step, positive samples are examined by tetraplex HA‐ and triplex NA‐specific RT‐qPCRs to differentiate the porcine subtypes H1, H3, N1 and N2. Within the HA subtype H1, lineages “av” (European avian‐derived), “hu” (European human‐derived) and “pdm” (human pandemic A/H1N1, 2009) are distinguished by RT‐qPCRs, and within the NA subtype N1, lineage “pdm” is differentiated. An RT‐PCR amplicon Sanger sequencing method of small fragments of the HA and NA genes is also proposed to safeguard against failure of multiplex RT‐qPCR subtyping.

**Conclusions:**

These new multiplex RT‐qPCR assays provide adequate tools for sustained SIV monitoring programmes in Europe.

## 1 Introduction

Influenza A virus (IAV) infections have a significant clinical impact on domestic swine populations. This is mainly related to acute respiratory disease which is frequently exacerbated by synergistic viral and bacterial co‐infections. Economic losses for the pork producing industry due to swine influenza virus (SIV) infections can be substantial.^1^, ^2^, ^3^

Swine are susceptible to infection from various IAV subtypes of both mammalian and avian origin. This is due to the expression of cellular glycan moieties (alpha 2‐6 or 2‐3‐linked terminal sialic acids, respectively) in the porcine upper respiratory tract that act as receptors for viral attachment by the haemagglutinin protein (HA) of both avian and mammalian IAV.^4^, ^5^, ^6^ Thus, simultaneous infection of porcine cells with mammalian/human and avian IAV is possible. Such double infections open possibilities for an exchange of genome segments between these viruses (reassortment) and progeny viruses with mixed parental genotypes (reassortants) potentially leading to the emergence of new phenotypic properties. For this reason, pigs have been dubbed *mixing vessels* of IAV.^7^, ^8^, ^9^, ^10^ Historically, the evolution of human and porcine influenza viruses has been intimately interlinked over at least one century in that new influenza virus reassortants have emerged in swine populations following trans‐species transmission of IAV from infected humans in the course of human IAV pandemics (so‐called reverse zoonotic transmission).^11^ However, direct infection of swine with IAV of purely avian origin has also occurred.^12^ In swine, some of these viruses swiftly established porcine‐adapted, independently circulating lineages which reassorted amongst each other. As a result of these processes, a variety of sublineages and genotypes of SIV is now prevalent in swine populations worldwide, but with some geographic restrictions,.^13^, ^14^ Some of these viruses may have zoonotic potential and can be retransmitted into the human population.^15^, ^16^, ^17^

Until 2009, the IAV subtypes H1N1, H1N2 and H3N2 circulated in domestic pigs at variable rates in Europe. The current enzootic Eurasian SIV H1N1 lineage is of purely avian origin and was introduced to pigs from unknown bird populations in the late 1970s. These viruses are referred to as “avian‐like” (av) H1N1 and are genetically and antigenically distinct from the “classical” (human 1918‐derived) H1N1 SIVs which continue to circulate in North America and some parts of Asia, but not in Europe.^13^, ^18^ Reassortment of viruses of the Eurasian H1N1av lineage with human seasonal H3N2 viruses gave rise to the porcine H3N2 lineage in which HA and NA are of human origin, while the other six genome segments are of avian descent.^14^ In 1994, an H1N2 reassortant (H1huN2) comprising the HA gene from a human seasonal H1N1 virus, the NA gene from H3N2 human‐like SIV and internal genes from H1N1 avian‐like SIV was first identified in the United Kingdom and subsequently detected in many further European countries.^19^ In 2003, a new H1N2 SIV reassortant virus was found in Danish pigs.^20^ This H1N2 virus comprised an avian‐like HA gene and an NA from contemporary circulating H3N2 SIV and have since been found in several other European countries.^18^ Apart from these dominant enzootic strains, several reassortants between these viruses have been described in Europe, but no sustained spread of such viruses has been reported as yet.^21^

Following the emergence of the human pandemic H1N1 2009 influenza A virus (H1N1pdm), a fourth IAV lineage entered swine populations globally. Pigs were found to be highly susceptible to this virus,^22^ and spread within swine populations, independent of human infections, is ongoing. An increasing amount of reassortants between porcine‐adapted enzootic IAV and the H1N1pdm virus have been described. Some of these reassortant lineages, such as the H1pdmN2 lineage reported from Germany, have been in regional circulation for several years now 23 and several retained the so‐called TRIG cassette of internal gene segments of the H1N1pdm virus and therefore may carry substantial zoonotic and even pandemic potential.^16^, ^21^

Despite coordinated European research efforts in surveillance of porcine IAV, such as the ESNIP consortium,^18^ data on the occurrence, spread and evolution of potentially zoonotic SIV in Europe remain fragmentary. This not only impedes the early detection of emerging SIV reassortants with increased zoonotic potential but may also interfere with the development and updating of efficacious influenza virus vaccines for use in pigs.^2^, ^3^ A prerequisite for efficient SIV surveillance programmes is sensitive and specific diagnostic methods which allow for high‐throughput analysis. Classically, SIV diagnosis is based on virus isolation in MDCK cells and serological subtype characterization by haemagglutination inhibition (HI) assays. However, this has recently been superseded by IAV‐generic real‐time RT‐PCRs (RT‐qPCR).^24^, ^25^, ^26^, ^27^ Molecular subtype characterization has, however, depended on time‐consuming sequencing of fragments of the HA and NA genes or on subtype‐specific, conventional RT‐PCRs.^28^, ^29^, ^30^

Here we present two multiplex RT‐qPCRs to facilitate the detection and differentiation of four and three lineages of HA and of NA gene segments, respectively, of SIV subtypes which currently cocirculate in European swine populations. In combination with a generic IAV‐specific duplex RT‐qPCR (enhanced by an internal control), we propose a diagnostic algorithm for a rapid, sensitive and lineage‐specific large‐scale monitoring of SIV infections in clinical samples from swine in Europe.

## 2 Material and Methods

### 2.1 Field samples, reference viruses

Lung tissue samples or nasal swabs derived from pigs with respiratory disease were obtained from swine holdings in several European countries in the frame of a passive monitoring programme. Samples were processed and analysed for SIV RNA by an M‐gene‐specific RT‐qPCR as detailed previously.^31^ Viral RNA of SIV‐positive samples was subtyped by molecular means using conventional pan‐HA and pan‐NA RT‐PCRs and amplicon Sanger sequencing.^32^, ^33^ In addition, virus isolation in MDCK cell cultures was attempted and isolates were subtyped by either full‐length nucleotide sequence analysis of the HA and NA genome segments as described elsewhere 34 or amplicon sequencing of short HA and NA fragments. influenza A viruses of other host species were retrieved from the reference collections at FLI (Friedrich‐Loeffler‐Institut, Greifswald‐Insel Riems, Germany) or RKI (Robert‐Koch‐Institut, Berlin, Germany) and used for RT‐qPCR validation purposes. Similarly, other porcine respiratory pathogens of viral and bacterial nature were used for assessing analytical specificity (Table S1).

### 2.2 Design of primers and probes

Primers and probes for subtype and/or lineage‐specific detection of HA and NA for use in multiplexed RT‐qPCRs were either selected from previously published assays (H1pdm: 35 N1 and N2 (Hoffmann et al.^36^ (2016) or manually selected (H1av, H1hu, H3, N1pdm) from alignments comprising full‐length HA and NA sequences of European SIV detected since year 2000; sequences were retrieved from the Influenza Research (IRD) and *Global Initiative on Sharing All Influenza Data* **(**GISAID) databases. Melting temperatures and basic properties of oligonucleotides were approximated using the online tool “Oligocalc”.^37^ Preliminary versions of the primer and probe sets were evaluated with European SIV isolates previously identified 18 in laboratories in France, Italy, United Kingdom, Denmark and Germany during the ESNIP3 concerted action. Based on these results, further rounds of optimizing oligonucleotide sequences were initiated. Table 1 lists the final sets of primers and probes for RT‐qPCRs (A) and conventional RT‐PCRs (B).

**Table 1:** Attributes of primers and probes employed in tetra‐ and triplex RT‐qPCRs (A) or in multiplex RT‐PCR (B) for the detection of HA and NA lineages, respectively, of swine influenza viruses currently circulating in Europe

Primer/Probe	Concentration	Sequence, labelling	Location	Product size	Reference sequence
(A)					
*Hemagglutinin*					
H1av					
H1av_Sw_Fn	20 pmol/rxn	gaaggrggatggacaggratra	1063–1084	134–139 bp	A/swine/Germany/SIV04/2008 (H1N1)
H1av_Sw_R1	10 pmol/rxn	actgagttcactttgttrytk	1176–1196		FN429078
H1av_Sw_R2	10 pmol/rxn	caattahtgarttcactttgttgy	1178–1201		
H1av_Sw_HEX_1	2,5 pmol/rxn	HEX‐tctggttaygcagcwgatmagaaaa‐BHQ1	1126–1150		
H1hu					
H1hu_Sw_F_2	10 pmol/rxn	ggatggtacggttaycayca	1090–1109	115–208 bp	A/swine/Kitzen/IDT6142/2007 (H1N2)
H1[N2]_Swine_Fw	10 pmol/rxn	gagggggrtggaccggaatgatagatgga[i]5tggttatcatca	1064–1109		GQ161145
H1hu_Sw_R_2	10 pmol/rxn	tttcgatcacagaattcacct	1184–1204		
H1[N2]_Swine_Rv	10 pmol/rxn	acctacagctgtgaattgagtgttcatyttntcg[i]5agagttcacct	1184–1233		
H1hu_Sw_ROX	2,5 pmol/rxn	ROX‐cagggwtctgghtatgctgcdgacc‐BHQ2	1120–1144		
H1pdm					
H1pdm_Fw	10 pmol/rxn	ctagtggtaccgagatatgca	793–813	88 bp	A/Germany‐MV/R26/2011 (pdmH1N1)
H1pdm_Rv	10 pmol/rxn	tattgcaatcgtggactggtgt	859–880		EPI356430
H1pdm_FAM	1 pmol/rxn	FAM‐cgcaatggaaagaaatgctggatctgg‐BHQ1	816–842		
H3					
H3_Sw_F2	10 pmol/rxn	ggggaccctcaytgtga	262–278	100 bp	A/swine/Bakum/8602/99 (H3N2)
H3_Sw_R2	10 pmol/rxn	actcyggcacrtcatahgg	343–361		EF409250
H3_Sw_Q670	2,5 pmol/rxn	Cy5‐ttgaacgcagcarrgctttcagca‐BHQ3	311–334		
*Neuraminidase*					
N1all					
N1.3_F	12,5 pmol/rxn	agrccttgyttctgggttga	1255–1274	126 bp	A/swine/Germany/SIV04/2008 (H1N1)
N1.3_R	12,5 pmol/rxn	accgtctggccaagacca	1363–1380		FN429079
AIV_N1.3_FAM	1,5 pmol/rxn	FAM‐atytggacyagtgggagcagcat‐BHQ1	1306–1328		
N1pdm					
N1pdm_FW_2	10 pmol/rxn	caacacttgggtaaatcaga	174–193	74 bp	A/Germany‐MV/R26/2011 (pdmH1N1)
N1pdm_RV_2	10 pmol/rxn	cggaaaccactgactgtc	230–247		EPI356429
N1pdm_SO_2	2,5 pmol/rxn	ROX‐catcagcaacaccaactttgctgct‐BHQ2	204–228		
N2all					
N2_1367F	15 pmol/rxn	agtctggtggacytcaaayag	1305–1325	116 bp	A/swine/Bakum/8602/99 (H3N2)
N2_1468R	15 pmol/rxn	ttgcgaaagcttatatagvcatga	1397–1420		EF409258
AIV_N2_1444_HEX	2,5 pmol/rxn	HEX‐ccatcaggccatgagcctgwwccata‐BHQ1	1357–1382		
(B)					
*panHA* a					
HA‐1057.1‐F	5 pmol/rxn	ggrgaatgccccaaataygt	952–971	180 bp	A/swine/Germany/SIV04/2008 (H1N1)
HA‐1057.2‐F	5 pmol/rxn	ggraratgccccagrtatgt	952–971		FN429078
HA‐1057.3‐F	5 pmol/rxn	ggrgaatgccccaartayat	952–971		
HA‐1232.1(555)‐R	5 pmol/rxn	ctgagtccgaacattgagttgctatgvtgrtawccatacca	1093–1115		
HA‐1232.2(555)‐R	5 pmol/rxn	ctgagtccgaacattgagttytgatgyctgaadccrtacca	1093–1115		
*panHA‐SIV*					
H1av					
PAN_H1av_DH_F	5 pmol/rxn	ggrgaatgccccaaataygt	952–971	180 bp	A/swine/Germany/SIV04/2008 (H1N1)
PAN_H1av_DH_R_M13	5 pmol/rxn	cgttgtcgatactggtacttgatgrtgatayccataccayc	1091–1113		FN429078
H1hu					
PAN_H1hu_DH_F	5 pmol/rxn	ggrgaatgccccaartaygt	952–971	180 bp	A/swine/Kitzen/IDT6142/2007 (H1N2)
PAN_H1hu_DH_R_M13	5 pmol/rxn	cgttgtcgatactggtaccygrtgatgataaccataccayc	1091–1113		GQ161145
H1pdm					
PAN_H1pdm_DH_F	5 pmol/rxn	gtgcyataaacaccagcctc	902–921	230 bp	A/Germany‐MV/R26/2011 (pdmH1N1)
PAN_H1pdm_DH_R_M13	5 pmol/rxn	cgttgtcgatactggtacttgatggtgataaccgtaccatc	1091–1113		EPI356430
H3					
PAN_H3_DH_F	5 pmol/rxn	aacargatcacatatggrgcatg	940–962	195 bp	A/swine/Bakum/8602/99 (H3N2)
PAN_H3_DH_R_M13	5 pmol/rxn	cgttgtcgatactggtacttgrtgyctgaaaccgtaccarc	1094–1116		EF409250
*panNA* a					
NA‐886.1‐F	10 pmol/rxn	atcgargartgytcntgyta	826–845	631 bp	A/swine/Germany/SIV04/2008 (H1N1)
NA‐886.2‐F	10 pmol/rxn	gtcgargartgytchtgbta	826–845		FN429079
AIVR‐1458‐R	10 pmol/rxn	gcagtatatcgcttgacaagtagaaacaagg	1426–1438		

^a^Gall et al.^33^, 2009.

rxn—volume reaction of 25 μL.

### 2.3 One‐step RT‐qPCR

The AgPath‐ID^TM^ One‐Step RT‐PCR kit (Ambion, Foster City, CA, USA) was used throughout. Thermocycling conditions on a Bio‐Rad CFX96 real‐time PCR detection system were optimized by adapting annealing time and temperature. These cycling conditions were found to be optimal for the generic M‐specific RT‐qPCR^38^:

- 10 minutes 45°C, 10 minutes 95°C, 42 cycles each of 15 seconds 95°C – 20 seconds 55°C – 30 seconds 72°C.

The cycling conditions for the multiplex HA‐ and NA‐specific RT‐qPCRs were found to be optimal at:

- 10 minutes 45°C, 10 minutes 95°C, 42 cycles each of 15 seconds 95°C – 30 seconds 58°C – 30 seconds 72°C.

The fluorescence data were collected during the annealing step of 55°C and 58°C, respectively, in all runs.

### 2.4 Conventional one‐step RT‐PCR

Superscript III One‐Step RT‐PCR kit with Platinum Taq polymerase (Invitrogen ^TM^ GmbH, Karlsruhe, Germany) was used for conventional RT‐PCR. Thermocycling conditions on an Analytik Jena Flex Cycler or a SensoQuest Labcycler were optimized by adapting annealing time and temperature. These cycling conditions were found to be optimal for the generic pan‐HA and pan‐NA 32, 33 RT‐PCRs:

- 30 minutes 50°C, 2 minutes 94°C, 45 cycles each of 30 seconds 94°C – 45 seconds 50°C – 45 seconds 68°C, final elongation 5 minutes 68°C.

The cycling conditions for an optimized version of the generic pan‐HA‐SIV RT‐PCR were found to be optimal at:

- 30 minutes 48°C, 2 minutes 94°C, 45 cycles each of 30 seconds 94°C – 30 seconds 55°C – 40 seconds 68°C, final elongation 2 minutes 68°C.

### 2.5 Sequencing

The subtype and lineage of SIV isolates and PCR‐positive field samples were determined by Sanger sequencing of RT‐PCR‐amplified fragments of the HA and NA gene segments using the pan‐HA and pan‐NA conventional RT‐PCRs described by Gall et al.^32^, ^33^ (2008, 2009) and further modified in this study. These PCRs generate an HA fragment of approximately 180 bp spanning the HA cleavage site and a 630‐bp fragment of the 5′ region of the NA gene segment. For selected SIV isolates, full‐length HA and NA sequences were obtained using RT‐PCRs described by Starick et al.^34^ (2011). Specific amplicons were purified from 1.5% agarose gels using a QIAquick gel extraction kit (Qiagen, Hilden, Germany) and Sanger‐sequenced using the RT‐PCR primers. The sequences were analysed on an ABI 310 sequencer, curated using the Chromas Lite^®^ software (http://www.technelysium.com.au/Chromas250Setup.exe) and assembled using the CAP3 programme (http://doua.prabi.fr/software/cap3).

### 2.6 Molecular sequence analyses

The IRD or GISAID databases were screened with BLASTN2 to identify closely related sequences of the pan‐HA and pan‐NA sequences.

## 3 Results

### 3.1 Assembly and analytical performance of subtype‐specific uniplex RT‐qPCRs for the detection and differentiation of HA and NA lineages of European SIV

Significant sequence variation exists within and between the HA and NA subtypes and lineages of SIV from Europe. Sequence stretches conserved within one lineage, yet distinctively different from other lineages were found to be rare even if only the 3′ termini of potential primers and/or the central region of probes were considered. Extensive in silico analysis showed that degeneration of primers at up to four positions or the use of more than two primers per PCR was inevitable to span the whole width of (published) sequence variation within a single lineage (Table 1). Primers selected by this strategy for the human‐derived H1huN2 lineage gave rise to false (cross‐subtype‐) priming and resulted in non‐specific amplification (data not shown). In an attempt to design more specific primers, dual priming oligonucleotides (DPO) were developed. Dual priming oligonucleotides primers are composed of a longer 5′ sequence stretch which serves as an anchor to firmly position the primer at the target site, and degenerate nucleotides can be included into this anchor region. The shorter 3′ region of the DPO primer of usually less than 12 nucleotides enforces highly specific hybridization within a fully matching target sequence. The two regions are separated by a stretch of five inosine residues.^30^, ^38^

RNA obtained from a panel of 20 IAV of subtype H1, H3, N1 and N2 of human, avian or porcine origin was used to evaluate different sets of primers and probes for their analytical specificity for different lineages of European SIV. Results shown in Table 2A prove that the sets finally chosen discriminated between the HA H1 lineages: (i) pandemic H1 (H1pdm), (ii) endemic avian‐origin porcine H1 (H1av) and (iii) H1 of the human‐derived H1N2 subtype (H1hu) and subtype porcine H3 (H3). The H1hu RT‐qPCR also detected the HA of pre‐2009 seasonal human H1N1 lineage represented by a historic (A/FM/1/1947) and a more recent human isolate (A/Germany‐NDS/14/2007). This (cross‐) reactivity is expected as the porcine H1hu lineage is derived as a reassortant from human seasonal H1N1 from the 1990s. No cross‐reactivity was observed with recent H1 and H3 IAV of avian origin.

**Table 2:** Analytical specificity of primers and probes for detection and differentiation of HA (A) and NA (B) lineages of swine influenza viruses from Europe. Results are based on uniplex RT‐qPCRs

(A) RT‐qPCRs targeting the haemagglutinin gene (isolates sorted by HA subtype/lineage)							
Isolate identification	Lineage		RT‐qPCR (Ct‐values)				
	HA	NA	M1.2	H1pdm	H1av	H1hu	H3
A/Germany/Regensburg/2009	H1pdm	N1pdm	23.6	22.5	neg.	neg.	neg.
A/Germany/NJH‐R26/2011	H1pdm	N1pdm	20.8	21.2	neg.	neg.	neg.
A/Swine/Germany/R75/2011	H1pdm	N2	18.3	17.9	neg.	neg.	neg.
A/Swine/Germany/R708/2010	H1pdm	N1av	22.2	19.6	neg.	neg.	neg.
A/Swine/Belzig/2/2001	H1av	N1av	18.7	neg.	17.6	neg.	neg.
A/Swine/Germany/R819/2010	H1av	N1av	21.9	neg.	25.0	neg.	neg.
A/Swine/Germany/R248/2010	H1av	N1av	23.9	neg.	22.5	neg.	neg.
A/Swine/Germany/R1738/2010	H1av	N1av	21.0	neg.	17.6	neg.	neg.
A/Swine/Germany/R369/2009	H1av	N2	23.6	neg.	24.3	neg.	neg.
A/Swine/Bakum/1893/2000	H1hu	N2	21.9	neg.	neg.	22.3	neg.
A/Swine/Germany/R757/2010	H1hu	N2	18.6	neg.	neg.	21.1	neg.
A/Swine/Germany‐NI/R3394/2009	H1hu	N1av	21.2	neg.	neg.	21.3	neg.
A/Fort Monmoth/1/1947	H1	N1	24.2	neg.	neg.	23.5	neg
A/Germany‐NDS/14/2007	H1	N1	29.0	neg.	neg.	32.9	neg.
A/White‐fronted goose/Germany/R482/2009	H1	N1	21.6	neg.	neg.	neg.	neg.
A/Wild duck/Germany/R30/2006	H1	N1	24.9	neg.	neg.	neg.	neg.
A/Swine/Bakum/909/1993	H3	N2	23.2	neg.	neg.	neg.	22.2
A/Swine/Germany/R96/2011	H3	N2	18.5	neg.	neg.	neg.	19.2
A/Swine/Germany/R76/2011	H3	N2	21.8	neg.	neg.	neg.	22.1
A/Waterfowl/Germany/2311/2009	H3	N8	24.7	neg.	neg.	neg.	neg.
A/Germany‐BW/23/2007	B	B	neg.	neg.	neg.	neg.	neg.
Tetraplex mixture				FAM	HEX	ROX	CY5

(B) RT‐qPCRs targeting the neuraminidase gene (isolates sorted by NA subtype/lineage)						
Isolate identification	Lineage		RT‐qPCR (Ct‐values)			
	HA	NA	M1.2	N1all	N1pdm	N2
A/Swine/Belzig/2/2001	H1av	N1av	14.5	16.3	neg.	neg.
A/Swine/Germany‐NI/R3394/2009	H1hu	N1av	15.3	14.6	neg.	neg.
A/Swine/DE/R708/2010	H1pdm	N1av	19.0	17.2	neg.	neg.
A/Swine/DE/R819/2010	H1av	N1av	16.3	16.5	neg.	neg.
A/Swine/DE/R248/2010	H1av	N1av	19.5	19.7	neg.	neg.
A/Swine/DE/R1738/2010	H1av	N1av	17.2	17.6	neg.	neg.
A/White‐fronted goose/Germany/R482/2009	H1	N1	18.0	19.9	neg.	neg.
A/Wild duck/Germany/R30/2006	H1	N1	16.2	17.6	neg.	neg.
A/Fort Monmoth/1/1947	H1	N1	15.1	15.3	neg.	neg.
A/Germany‐NDS/14/2007	H1	N1	20.6	19.0	neg.	neg.
A/Germany/Regensburg/2009	H1pdm	N1pdm	18.7	20.0	20.0	neg.
A/Germany/NJH‐R26/2011	H1pdm	N1pdm	17.9	18.2	19.1	neg.
A/Swine/Bakum/1893/2000	H1hu	N2	16.5	neg.	neg.	23.3
A/Swine/Germany/R757/2010	H1hu	N2	15.0	neg.	neg.	17.0
A/Swine/Germany/R75/2011	H1pdm	N2	13.7	neg.	neg.	19.1
A/Swine/Germany/R369/2009	H1av	N2	18.8	neg.	neg.	18.3
A/Swine/Bakum/909/1993	H3	N2	18.8	neg.	neg.	19.5
A/Swine/Germany/R96/2011	H3	N2	15.0	neg.	neg.	15.2
A/Swine/Germany/R76/2011	H3	N2	18.1	neg.	neg.	19.3
A/Waterfowl/Germany/2311/2009	H3	N8	neg.	neg.	neg.	neg.
A/Germany‐BW/23/2007	B	B	neg.	neg.	neg.	neg.
Triplex mixture				FAM	ROX	HEX

neg.: Negative.

In contrast to HA‐specific PCRs, no host‐specific differentiation of N1 and N2 subtypes was attempted. Instead, broadly reacting RT‐qPCRs for subtypes N1 (N1all) and N2 were established (Hoffmann et al.; FLI, Greifswald‐Insel Riems, Germany, submitted for publication) and tested favourably with the reference RNA panel (Table 2B). In addition, another RT‐qPCR specifically detecting the N1 of the human pandemic/2009 H1N1 lineage (N1pdm) was developed. Thus, N1pdm‐positive viruses gave positive results with both the N1all and the N1pdm RT‐qPCRs (Table 2B).

None of the primer and probe sets gave false‐positive results when tested with IAV subtypes H1 and H3 of avian origin (Table 2).

Non‐specific reactivity of these PCRs with other porcine viral or bacterial respiratory pathogens as listed in Table S1 was excluded.

### 3.2 Assembly and analytical performance of tetraplex (4plex) and triplex (3plex) RT‐qPCRs for the detection of haemagglutinin and neuraminidase sequences of European SIV

The HA‐ and NA‐specific probes were labelled with different colours: FAM—H1pdm, HEX—H1av, ROX—H1hu, Cy5—H3; FAM—N1, ROX—N1pdm, HEX—N2. Primers and probes for each of the four HA and for the three NA targets were mixed generating a tetra‐ and a triplex amplification assay, respectively. Series of 10‐fold diluted RNA of eight representative virus isolates (two for each porcine HA subtype/lineage) were used to check for possible impact on the RT‐qPCR performance that may result from mixing the sets of primer and probes. No impairment of performance was noticed. Thus, detection continued to be highly lineage‐ and/or subtype‐specific and no cross‐reaction was evident even in samples with very high virus loads (data not shown). Comparison of Cq values of serial 10‐fold RNA dilutions with results of the generic M‐specific RT‐qPCR was used to assess the relative analytical sensitivity of the multiplex RT‐qPCRs. In general, relative detection limits of the multiplex RT‐qPCRs were slightly lower (up to 3 Cq values) than those of the corresponding M RT‐qPCR (Table 3).

**Table 3:** Relative sensitivity of tetraplex HA‐ and triplex NA‐specific RT‐qPCRs compared with IAV‐generic M‐specific amplification

(A)								
RNA dilution	H1pdm		H1av		H1hu		H3	
	M	4plex	M	4plex	M	4plex	M	4plex
0	24.3	22.3	24.5	23.1	22.5	22.9	22.6	21.3
−1	27.2	25.3	26.1	26.6	25.6	26.4	26.1	24.7
−2	30.5	28.9	29.4	30.1	29.1	30.4	29.2	28.4
−3	34.2	33.3	32.8	34.7	32.1	33.8	32.5	33.0
−4	36.6	39.8	35.4	neg.	35.4	neg.	36.1	neg.
−5	neg.	neg.	neg.	neg.	neg.	neg.	neg.	neg.
−6	neg.	neg.	neg.	neg.	neg.	neg.	neg.	neg.

**(B)**						
RNA dilution	N1aII		N1pdm		N2	
	M	3plex	M	3plex	M	3plex
0	24.5	24.8	24.3	26.0	24.3	25.2
−1	26.1	28.4	27.2	30.0	27.5	28.4
−2	29.4	31.8	30.5	33.6	31.0	32.0
−3	32.8	35.4	34.2	37.2	33.8	34.6
−4	35.4	39.1	36.6	39.7	37.8	38.6
−5	neg.	neg.	neg.	neg.	neg.	neg.
−6	neg.	neg.	neg.	neg.	neg.	neg.

neg.: Negative.

Serial 10‐fold dilutions of SIV RNA were examined by M‐ and tetraplex HA‐ (A) and triplex NA‐specific (B) RT‐qPCRs.

H1pdm, N1pdm—A/Germany‐MV/R26/2011; H1av N1av—A/Swine/Germany/AR1738/2010; H1huN2—A/Swine/Germany/AR1141/2014; H3N2—A/Swine/Germany/R655/2012.

### 3.3 Detection of SIV double infections by HA‐specific tetraplex and NA‐specific triplex RT‐qPCRs

Infection of the same cell by more than one IAV geno‐ or subtype is the prerequisite of reassortment. The possibility to detect influenza virus co‐infections in the same clinical samples is of interest in surveillance programmes. In order to mimic double infections, RNA of viruses of four different porcine HA and three NA subtypes/lineages, respectively, was mixed to represent all possible combinations (Table 4). The RNA of two viruses was mixed in approximately equal amounts, in 95:5 and in 5:95 to mimic an up to 20‐fold difference in RNA content (Table 4). Cq values of the M‐specific RT‐qPCR were used to normalize the concentration of viral RNA, assuming that this PCR‐amplified viral RNA of the different lineages with similar efficacy. In all cases, both HA and NA (if disparate) targets present in the sample were detected and discerned in all mixtures. Again, no cross‐reactivity to lineages not represented in the sample mix was evident.

**Table 4:** Detection of mixtures of different European SIV HA and NA lineages by tetraplex HA‐ (A) and triplex NA‐specific (B) RT‐qPCRs

(A)					
Virus	RNA percentage	4plex RT‐qPCR (Ct‐values)			
		H1pdm	H1av	H1hu	H3
H1pdm/H1av	95/5	26.1	35.8	neg.	neg.
H1pdm/H1av	50/50	27.1	28.8	neg.	neg.
H1pdm/H1av	5/95	31.0	27.6	neg.	neg.
H1pdm/H3	95/5	25.9	neg.	neg.	32.3
H1pdm/H3	50/50	27.0	neg.	neg.	25.2
H1pdm/H3	5/95	31.2	neg.	neg.	24.1
H1pdm/H1hu	95/5	26.0	neg.	30.4	neg.
H1pdm/H1hu	50/50	27.0	neg.	25.4	neg.
H1pdm/H1hu	5/95	31.5	neg.	24.6	neg.
H1hu/H3	95/5	neg.	neg.	24.7	30.8
H1hu/H3	50/50	neg.	neg.	25.5	25.6
H1hu/H3	5/95	neg.	neg	30.6	24.3
H1hu/H1av	95/5	neg.	27.7	27.7	neg.
H1hu/H1av	50/50	neg.	28.7	25.6	neg.
H1hu/H1av	5/95	neg.	33.3	24.4	neg.
H1av/H3	95/5	neg.	26.9	neg.	30.5
H1av/H3	50/50	neg.	28.2	neg.	25.5
H1av/H3	5/95	neg.	33.3	neg.	24.1

(B)				
Virus	RNA percentage	3plex RT‐qPCRs (Ct‐values)		
		N1all	N1pdm	N2
N1pdm/N1av	95/5	26.3	26.6	neg.
N1pdm/N1av	50/50	26.4	27.7	neg.
N1pdm/N1av	5/95	26.5	31.3	neg.
N1pdm/N2 (H3)	95/5	26.5	26.2	29.3
N1pdm/N2 (H3)	50/50	27.5	27.6	26.7
N1pdm/N2 (H3)	5/95	30.6	31.6	26.2
N1av/N2 (H3)	95/5	26.0	neg.	29.5
N1av/N2 (H3)	50/50	27.0	neg.	26.9
N1av/N2 (H3)	5/95	29.8	neg.	25.8

neg.: Negative.

For tetraplex HA RT‐qPCR mixtures, RNA extracted from the following SIV isolates was mixed: H1N1pdm—A/Swine/Germany/1760/2014; H1N1av—A/Swine/Germany/AR1738/2010; H1huN2—A/Swine/Germany/AR1146/2014; H3N2—A/Swine/Germany/R655/2012.

Triplex NA RT‐qPCR mixtures consisted of RNA extracted from the following SIV isolates: H1N1pdm—A/Swine/Germany/1759/2014; H1N1av—A/Swine/Germany/AR1696/2014; H3N2—A/Swine/Germany/R655/2012.

The relative amount of RNA was normalized to a dilution that generated a Cq value of 25‐26 using the M‐specific RT‐qPCR for all isolates. Then, the mixtures were prepared to yield the percentage values indicated in the second column of the tables.

### 3.4 Diagnostic performance of the HA‐specific tetraplex‐ and NA‐specific triplex RT‐qPCRs

A total of 27 SIV isolates obtained in MDCK cell cultures and 27 porcine nasal swabs which had tested positive for IAV RNA by M‐specific RT‐qPCR were derived from European swine populations with overt respiratory symptoms. In addition, 27 porcine nasal swab samples that tested negative for IAV RNA were examined. The multiplex RT‐qPCRs did not yield positive signals for any of the IAV‐negative samples (data not shown). Subtyping results obtained by the newly developed multiplex RT‐qPCRs for M‐positive isolates and samples were compared to those generated by conventional RT‐PCR and amplicon Sanger sequencing. For this purpose, pan‐HA and pan‐NA RT‐PCRs were used as published by Gall et al.^32^, ^33^ (2008, 2009).

Tables 5A/B compares subtyping results by HA and NA amplicon sequencing and by HA and NA multiplex RT‐qPCRs for clinical SIV isolates. Test of all RNAs extracted from isolates indicated high virus loads as indicated by the M‐specific Cq values (range 16–23). All the HA and NA subtypes were unambiguously assignable by multiplex RT‐qPCRs with the exception of three isolates for which no signal was obtained in the HA tetraplex RT‐qPCR (Table 5A). Full‐length sequences of genome segment 4 encoding the HA protein were generated from these three isolates and the sequences compared to the oligonucleotide sequence of the primers and probe used in the subtyping RT‐qPCRs. This analysis revealed a considerable number of mismatches (Fig. S1a). Also, the products of the tetraplex RT‐qPCR of these isolates were examined by gel electrophoresis (Fig. S1b), but no specific amplicons were detected. In two cases (AR1123/15 and 2196/15, Table 5A), the multiplex RT‐qPCRs detected the presence of a mixture of H1av and H1hu subtypes in the same isolate.

**Table 5:** Haemagglutinin and neuraminidase subtyping of RNA of European SIV extracted from isolates (A, B) or from clinical samples (C, D) by multiplex RT‐qPCR (this study) or conventional multiplex RT‐PCR and Sanger sequencing

(A) SIV isolates, HA											
Isolate identification			RT‐qPCR (Ct‐values)					RT‐PCR/sequencing			Harmonized diagnosis
Sample	Country	Subtype	M1.2	H1pdm	H1av	H1hu	H3	HA Full‐length	panHA	panHA‐SIV	HA
AR 1279/15	ES	H1avN1av	16.8	neg.	34.4	neg.	neg.		H1av		H1av
AR 1340/15	FR	H1avN1av	22.8	neg.	23.1	neg.	neg.		H1av		H1av
AR 1348/15	FR	H1avN1av	16.0	neg.	19.1	neg.	neg.		H1av		H1av
AR 2618/15	BE	H1avN1av	16.1	neg.	23.6	neg.	neg.		Questionable	H1av	H1av
AR 2754/15	FR	H1avN1av	18.6	neg.	18.4	neg.	neg.		Failed	H1av	H1av
AR 2778/15	FR	H1avN1av	17.3	neg.	19.1	neg.	neg.		Failed	H1av	H1av
AR 2783/15	FR	H1avN1av	18.6	neg.	20.1	neg.	neg.		H1av		H1av
AR 2667/15	DK	**H1avN2**	27.7	neg.	19.0	neg.	neg.		Questionable	H1av	H1av
AR 2759/15	UK	**H1avN2**	23.3	neg.	neg.	neg.	neg.		Failed	H1av	H1av
AR 3067/15	DK	**H1avN2**	14.7	neg.	17.2	neg.	neg.		Questionable	H1av	H1av
AR 1123/15	FR	**H1av/hu**	20.6	neg.	22.3	22.3	neg.		H1av/hu		H1av/hu
AR 2196/15	FR	**H1av/hu**	18.2	neg.	20.0	26.3	neg.		Failed	H1av	H1av/hu
AR 1359/15	FR	H1huN2	18.0	neg.	neg.	neg.	neg.		H1hu		H1hu
AR 1372/15	ES	H1huN2	16.2	neg.	neg.	22.1	neg.		H1hu		H1hu
AR 2023/15	FR	H1huN2	19.0	neg.	neg.	30.6	neg.		H1hu		H1hu
AR 2829/15	DE	H1huN2	16.3	neg.	neg.	26.2	neg.	H1hu			H1hu
AR 647/15	NL	**H1huN1av**	18.7	neg.	neg.	20.5	neg.		H1hu		H1hu
AR 2056/15	NL	**H1huN1av**	19.1	neg.	neg.	20.2	neg.		H1hu		H1hu
AR 2082/15	NL	**H1huN1av**	19.7	neg.	neg.	20.5	neg.		H1hu		H1hu
AR 1855/15	DK	H1pdmN1pdm	17.9	18.9	neg.	neg.	neg.	H1pdm			H1pdm
AR 2675/15	FR	H1pdmN1pdm	18.3	21.1	neg.	neg.	neg.		Questionable	H1pdm	H1pdm
AR 2749/15	DE	**H1pdmN2**	17.4	19.9	neg.	neg.	neg.	H1pdm			H1pdm
AR 531/15	NL	H3N2	19.6	neg.	neg.	neg.	18.1		Questionable	H3	H3
AR 543/15	NL	H3N2	20.2	neg.	neg.	neg.	17.6		H3		H3
AR 1203/15	BE	H3N2	22.6	neg.	neg.	neg.	20.2		H3		H3
AR 1204/15	BE	H3N2	19.1	neg.	neg.	neg.	18.1		H3		H3
AR 3179/15	NL	H3N2	13.2	neg.	neg.	neg.	neg.		H3		H3

(B) SIV isolates, NA									
Isolate identification			RT‐qPCR (Ct‐values)				RT‐PCR/sequencing		Harmonized diagnosis
Sample	Country	Subtype	M1.2	N1all	N1pdm	N2	NA Full‐length	panNA	NA
AR 1279/15	ES	H1avN1av	16.8	26.1	neg.	neg.		N1	N1av
AR 1340/15	FR	H1avN1av	22.8	22.8	neg.	neg.		N1	N1av
AR 1348/15	FR	H1avN1av	16.0	17.9	neg.	neg.		N1	N1av
AR 2618/15	BE	H1avN1av	16.1	16.4	neg.	neg.		N1	N1av
AR 2754/15	FR	H1avN1av	18.6	17.4	neg.	neg.		N1	N1av
AR 2778/15	FR	H1avN1av	17.3	19.7	neg.	neg.		N1	N1av
AR 2783/15	FR	H1avN1av	18.6	21.2	neg.	neg.		N1	N1av
AR 2667/15	DK	**H1avN2**	27.7	neg.	neg.	19.4		N2	N2
AR 2759/15	UK	**H1avN2**	23.3	neg.	neg.	25.3		N2	N2
AR 3067/15	DK	**H1avN2**	14.7	neg.	neg.	19.4		N2	N2
AR 1123/15	FR	**H1av/hu**	20.6	20.1	neg.	neg.		N1	N1av
AR 2196/15	FR	**H1av/hu**	18.2	17.7	neg.	neg.		N1	N1av
AR 1359/15	FR	H1huN2	18.0	neg.	neg.	18.4		Failed	N2
AR 1372/15	ES	H1huN2	16.2	neg.	neg.	17.6		N2	N2
AR 2023/15	FR	H1huN2	19.0	neg.	neg.	31.1		N2	N2
AR 2829/15	DE	H1huN2	16.3	neg.	neg.	17.6	N2		N2
AR 647/15	NL	**H1huN1av**	18.7	17.9	neg.	neg.		N1	N1av
AR 2056/15	NL	**H1huN1av**	19.1	18.7	neg.	neg.		N1	N1av
AR 2082/15	NL	**H1huN1av**	19.7	18.5	neg.	neg.		N1	N1av
AR 1855/15	DK	H1pdmN1pdm	17.9	20.5	18.4	neg.	N1pdm		N1pdm
AR 2675/15	FR	H1pdmN1pdm	18.3	19.9	19.1	neg.		N1pdm	N1pdm
AR 2749/15	DE	**H1pdmN2**	17.4	neg.	neg.	21.1	N2		N2
AR 531/15	NL	H3N2	19.6	neg.	neg.	25.3		N2	N2
AR 543/15	NL	H3N2	20.2	neg.	neg.	32.2		N2	N2
AR 1203/15	BE	H3N2	22.6	neg.	neg.	28.9		N2	N2
AR 1204/15	BE	H3N2	19.8	neg.	neg.	25.4		N2	N2
AR 3179/15	NL	H3N2	13.2	neg.	neg.	13.9		N2	N2

(C) Clinical samples, HA										
Sample identification			RT‐qPCR (Ct‐values)					RT‐PCR/sequencing		Harmonized diagnosis
Sample	Country	Subtype	M1.2	H1pdm	H1av	H1hu	H3	panHA	panHA‐SIV	HA
AR 849/15	FR	**H1avN1av**	22.0	neg.	27.2	neg.	neg.	Failed	H1av	**H1av**
AR 1350/15	FR	**H1avN1av**	29.9	neg.	32.5	neg.	neg.	Failed	H1av	**H1av**
AR 1520/15	FR	**H1avN1av**	25.8	neg.	22.5	neg.	neg.	H1av		**H1av**
AR 1766/15	DE	**H1avN1av**	20.1	neg.	21.0	neg.	neg.	H1av		**H1av**
AR 2089/15	NL	**H1avN1av**	35.0	neg.	25.8	neg.	neg.	Failed	Failed	**H1av**
AR 2297/15	FR	**H1avN1av**	29.2	neg.	32.9	neg.	neg.	Failed	Failed	**H1av**
AR 2435/15	ES	**H1avN1av**	32.3	neg.	32.2	neg.	neg.	Failed	Failed	**H1av**
AR 3298/15	FR	**H1avN1av**	27.7	neg.	28.2	neg.	neg.	Failed	H1av	**H1av**
AR 682/15	ES	H1avN2	26.5	neg.	29.5	neg.	neg.	Failed	H1av	H1av
AR 856/15	ES	H1avN2	28.5	neg.	31.5	neg.	neg.	Failed	H1av	H1av
AR 1051/15	ES	H1avN2	30.6	neg.	38.3	neg.	neg.	Failed	Failed	H1av
AR 1992/15	DK	H1avN2	34.8	neg.	35.0	neg.	neg.	Failed	Failed	H1av
AR 1996/15	DK	H1avN2	26.8	neg.	24.3	neg.	neg.	Failed	H1av	H1av
AR 2662/15	DK	H1avN2	34.5	neg.	25.9	neg.	neg.	Failed	H1av	H1av
AR 2975/15	DK	H1avN2	24.6	neg.	28.5	neg.	neg.	Failed	Failed	H1av
AR 482/15	NL	**H1huN2**	24.4	neg.	neg.	28.9	neg.	Failed	H1hu	**H1hu**
AR 1355/15	FR	**H1huN2**	26.8	neg.	neg.	30.3	neg.	Failed	H1hu	**H1hu**
AR 3002/15	BE	**H1huN2**	22.9	neg.	neg.	26.2	neg.	Failed	H1hu	**H1hu**
AR 3437/15	NL	**H1huN2**	25.9	neg.	neg.	27.9	neg.	Failed	H1hu	**H1hu**
AR 2061/15	NL	H1huN1av	26.5	neg.	neg.	21.2	neg.	H1hu		H1hu
AR 1843/15	DK	**H1pdmN1pdm**	25.3	25.2	neg.	neg.	neg.	Failed	H1pdm	**H1pdm**
AR 2652/15	DK	**H1pdmN1pdm**	24.9	24.7	neg.	neg.	neg	Failed	H1pdm	**H1pdm**
AR 3404/15	FR	**H1pdmN1pdm**	29.0	27.4	neg.	neg.	neg.	Failed	H1pdm	**H1pdm**
AR 112/16	FR	**H1pdmN1pdm**	25.3	24.1	neg.	neg.	neg.	Failed	H1pdm	**H1pdm**
AR 2906/15	UK	H1pdmN1av	34.7	35.6	neg.	neg	neg.	Failed	Failed	H1pdm
AR 544/15	NL	**H3N2**	26.0	neg.	neg.	neg.	34.2	Failed	H3	**H3**
AR 220/16	NL	**H3N2**	22.3	neg	neg.	neg.	24.3	H3		**H3**

(D) Clinical samples, NA								
Sample identification			RT‐qPCR (Ct‐values)				RT‐PCR/sequencing	Harmonized diagnosis
Sample	Country	Subtype	M1.2	N1all	N1pdm	N2	panNA	NA
AR 849/15	FR	**H1avN1av**	22.0	22.1	neg.	neg.	N1	**N1av**
AR 1350/15	FR	**H1avN1av**	29.9	29.4	neg.	neg.	N1	**N1av**
AR 1520/15	FR	**H1avN1av**	25.8	24.6	neg.	neg.	N1	**N1av**
AR 1766/15	DE	**H1avN1av**	20.1	19.3	neg.	neg.	N1	**N1av**
AR 2089/15	NL	**H1avN1av**	35.0	32.6	neg.	neg.	Failed	**N1av**
AR 2297/15	FR	**H1avN1av**	29.2	29.0	neg.	neg.	N1	**N1av**
AR 2435/15	ES	**H1avN1av**	32.3	30.6	neg.	neg.	Failed	**N1av**
AR 3298/15	FR	**H1avN1av**	27.7	26.9	neg.	neg.	N1	**N1av**
AR 682/15	ES	H1avN2	26.5	neg.	neg.	30.6	N2	N2
AR 856/15	ES	H1avN2	28.5	neg.	neg.	31.7	N2	N2
AR 1051/15	ES	H1avN2	30.6	neg.	neg.	32.1	Failed	N2
AR 1992/15	DK	H1avN2	34.8	neg.	neg.	35.6	Failed	N2
AR 1996/15	DK	H1avN2	26.8	neg.	neg.	25.4	Failed	N2
AR 2662/15	DK	H1avN2	34.5	neg.	neg.	27.9	N2	N2
AR 2975/15	DK	H1avN2	24.6	neg.	neg.	26.0	N2	N2
AR 482/15	NL	**H1huN2**	24.4	neg.	neg.	27.4	N2	**N2**
AR 1355/15	FR	**H1huN2**	26.8	neg.	neg.	28.1	N2	**N2**
AR 3002/15	BE	**H1huN2**	22.9	neg.	neg.	23.2	N2	**N2**
AR 3437/15	NL	**H1huN2**	25.9	neg.	neg.	25.9	N2	**N2**
AR 2061/15	NL	H1huN1av	26.5	23.6	neg.	neg.	N1	N1av
AR 1843/15	DK	**H1pdmN1pdm**	25.3	26.2	25.4	neg.	N1pdm	**N1pdm**
AR 2652/15	DK	**H1pdmN1pdm**	24.9	23.3	22.2	neg.	N1pdm	**N1pdm**
AR 3404/15	FR	**H1pdmN1pdm**	29.0	28.5	26.8	neg.	N1pdm	**N1pdm**
AR 112/16	FR	**H1pdmN1pdm**	25.3	26.1	23.4	neg.	N1pdm	**N1pdm**
AR 2906/15	UK	H1pdmN1av	34.7	34.8	neg.	neg.	Failed	N1av
AR 544/15	NL	**H3N2**	26.0	neg.	neg.	31.5	N2	**N2**
AR 220/16	NL	**H3N2**	22.3	neg.	neg.	21.9	N2	**N2**

neg.: Negative.

“Sample number” indicates the identity of a sample (AR NNNN) and the year of sampling (/YR).

Bold‐face subtype signal HA/NA reassortants.

“failed” and “questionable” indicate that a no or only very short nucleotide sequences were generated.

“Harmonized results” identifies the subtype pattern as defined by combined results from RT‐qPCRs and RT‐PCR/sequencing.

Haemagglutinin and NA subtypes could be assigned to all of the clinical samples when tested by the multiplex RT‐qPCRs (Table 5C/D); however, this sample type revealed a greater variation in viral loads (range 21–35) compared with the cell‐grown isolates.

Amplicon sequencing based on the pan‐HA/pan‐NA RT‐PCRs described by Gall et al.^32^, ^33^2008/2009 was used to verify subtyping results generated by multiplex RT‐qPCRs. However, 9 of 27 isolates and 23 of 27 clinical samples failed to yield reliable HA sequences (Table 5, “questionable”) or failed to produce any amplicons at all (Table 5, “failed”). The NA subtyping was affected at a much lower rate (one isolate and six clinical samples failed). These results prompted us to establish updated primers of the pan‐HA PCR that provided a better match with European SIV and yielded enhanced amplification sensitivity. Modified primers are listed in Table 1B. Using the updated pan‐HA primer set in a multiplex RT‐PCR (termed “pan‐HA‐SIV”), it was possible to sequence amplicons for all 9 isolates which had failed before, and for 16 of the 23 failed clinical samples (Table 5).

Finally, a harmonized diagnosis was made by combining the results of the multiplex RT‐qPCR and the RT‐PCR/amplicon sequencing: in all cases for which results for both methods were available, fully matching subtyping results were obtained for both HA and NA (Table 5A–D). For seven HA samples for which also the updated primer set failed to produce an amplicon and for one failed NA sample, multiplex RT‐qPCRs were able to assign a subtype.

## 4 Discussion

Despite the fact that zoonotic and even pandemic potential in SIV has been noticed repeatedly, no sustained government‐administrated surveillance programmes currently operate in Europe, with the sole exception of France, that target SIV in swine populations.^18^ Technical problems and high costs associated with previously established SIV detection and subtyping tools may at least in part be related to the diffidence of embarking on monitoring on a larger scale. This discrepancy was taken as an incentive to develop more productive SIV subtyping tools.

Real‐time PCR is an established method in most routine diagnostic laboratories. The use of multiplex assays allows time‐efficient and cost‐effective examination of several parameters in the same tube.^40^ The ultimate goal of this study was to distinguish HA and NA lineages of SIV circulating in Europe and was achieved by the establishment of two multiplex RT‐qPCRs. Selection of primers and probes proved to be difficult as sequence stretches that are broadly conserved within one lineage (inclusivity) but distinct to all other ones (exclusivity) are scarce, especially in the HA genome segment. Therefore, primers and probes had to be constructed with a number of degenerate positions. In addition, mixtures of up to four primers for the same target had to be used. Finally, for the distinction of the human‐derived HA H1 lineage of SIV (H1hu), a double‐priming strategy had to be implemented. Validation of the analytical specificity of primers and probes in the uni‐ and multiplex formats confirmed their lineage‐specific reactivity, and therefore, detection of double infections with different lineages in the same sample was possible with high reliability. Significant advantages of the multiplex RT‐qPCRs over the original amplicon sequencing system were evident, especially for direct subtyping from clinical samples. Even samples with marginal RNA contents (Cq values up to 35) could be subtyped by RT‐qPCRs but failed to react even in the updated amplicon sequencing system, pan‐HA‐SIV.

When analysing RNA from SIV isolates, limits of the inclusivity of the multiplex RT‐qPCR became evident: despite the presence of high RNA loads in the samples (as evidenced by M RT‐qPCR), one isolate each of the H1av, H1hu and H3 lineage was not detected by the tetraplex HA‐specific RT‐qPCR; however, amplicon sequencing succeeded in all three cases to identify the HA subtype. Full‐length sequencing of the HA open reading frame of these isolates revealed a substantial number of mutated positions affecting the binding of both primers and probes (Table 6) which likely prevented amplification (Figs S1 and S2). A constant control and timely adaptation of oligonucleotides in the multiplex RT‐qPCRs is essential.

**Table 6:** Mismatches of primers and probes for the three SIV isolates which remained negative in the tetraplex HA RT‐qPCR (Table 5A)

Isolate identification			Primer	Mismatches
Sample	Country	Subtype		
AR 2759/15	UK	H1avN2	H1av_Sw_Fn	2
			H1av_Sw_R1	8
			H1av_Sw_R2	7
			H1av_Sw_HEX_1	4
AR 1359/15	FR	H1huN2	H1hu_Sw_F_2	1
			H1[N2]_Swine_Fw	2
			H1hu_Sw_R_2	3
			H1[N2]_Swine_Rv	1
			H1hu_Sw_ROX	2
AR 3179/15	NL	H3N2	H3_Sw_F2	2
			H3_Sw_R2	2
			H3_Sw_Q670	2

Full‐length HA sequences were established of the genome segment 4 of these viruses to align oligonucleotide sequences of primers and probes. Sequences are available from the Epiflu database at GISAID: EPI734316, EPI734466 and EPI734318.

While the isolates and clinical samples used here for validation purposes only represent a small fraction of the ongoing monitoring project, the comparatively large fraction of HA/NA reassortants is notable (Table 5, bold‐face samples). Increasing diversity of genotypes and HA/NA reassortant patterns, especially within subtype H1, has previously been noticed.^18^, ^21^, ^31^, ^41^ No reassortants were detected here for the H3 subtype.

**Figure 1:**
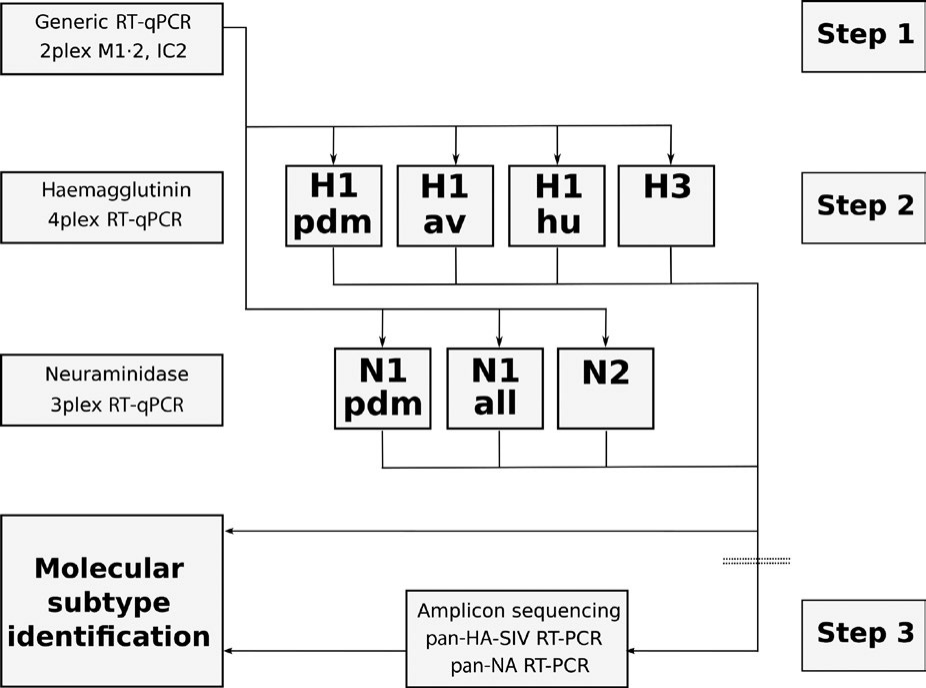
Proposed diagnostic algorithm for detection and subtyping of swine influenza viruses in Europe based on one‐step multiplex RT‐qPCRs. *Step 1* depicts an IAV‐generic one‐step RT‐qPCR, for example targeting the M gene segment; an internal control (IC2) is essentially included in this duplex RT‐qPCR. For IAV RNA‐positive samples, subtyping is attempted in *step 2* employing the one‐step tetraplex HA‐ and the triplex NA‐specific RT‐qPCRs developed in this study. Only for cases in which HA or NA subtype/lineage cannot be assigned by these RT‐qPCRs, *step 3* is required and HA and/or NA amplicons need to be generated by conventional one‐step RT‐PCR for Sanger amplicon sequencing and BLAST searches to finalize subtyping

Based on the results of this study, we propose an algorithm, depicted in Figure 1, for the detection and subtyping of porcine influenza viruses currently circulating in Europe. Any sample that tested positive in a generic influenza A virus RT‐qPCR (e.g. the modified Spackman M PCR enhanced with an internal control) is subjected in a second step to tetraplex HA‐ and triplex NA‐specific RT‐qPCRs which enable the delineation of four HA and three NA lineages/subtypes, respectively, of European SIV. In case either of these multiplex PCRs yields negative results, the subtype has to be determined by amplicon sequencing. The updated conventional pan‐HA‐SIV and the original pan‐NA RT‐PCRs previously developed by Gall et al.^33^ (2009) can be used as a complementing amplicon sequencing system. Alternatively, other published RT‐PCR protocols for the generation of lineage‐specific SIV HA and NA amplicons might be used.^28^

The newly developed multiplex RT‐qPCRs provide basic tools for a sustained monitoring programme of swine influenza in Europe. In addition, virus isolation on selected samples is required to allow further antigenic, in‐depth genetic and biological characterizations of circulating virus strains.
