# Supplementary figures and images for: Rapid detection and subtyping of European swine influenza viruses in porcine clinical samples by haemagglutinin‐ and neuraminidase‐specific tetra‐ and triplex real‐time RT‐PCRs
Source: Influenza Other Respir Viruses. 2016 Aug 9;10(6):504–17. doi: 10.1111/irv.12407 (PMC5059951; doi:10.1111/irv.12407)

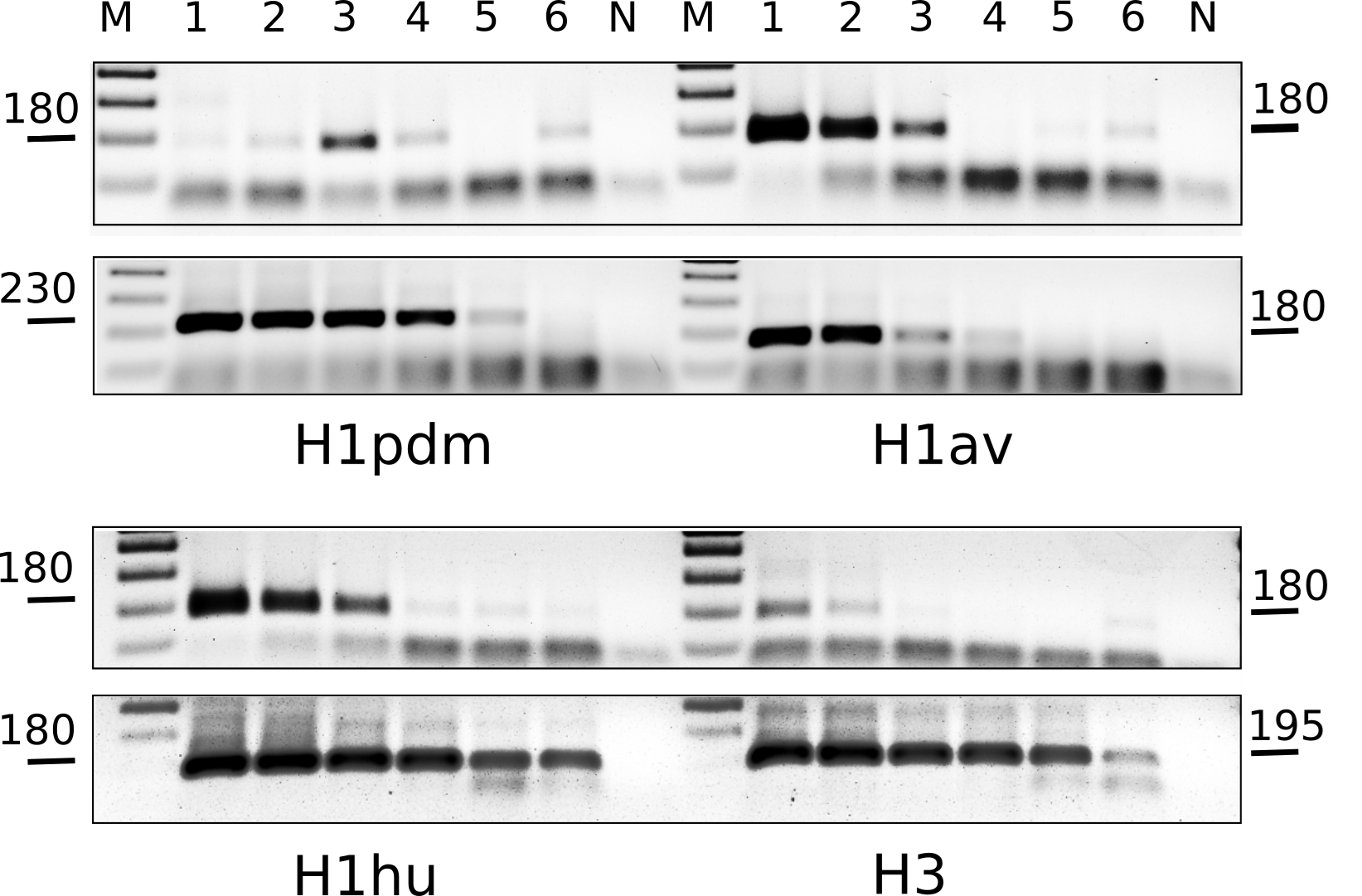

Supplement: Supplementary file 1 [file IRV-10-504-s001.tif]

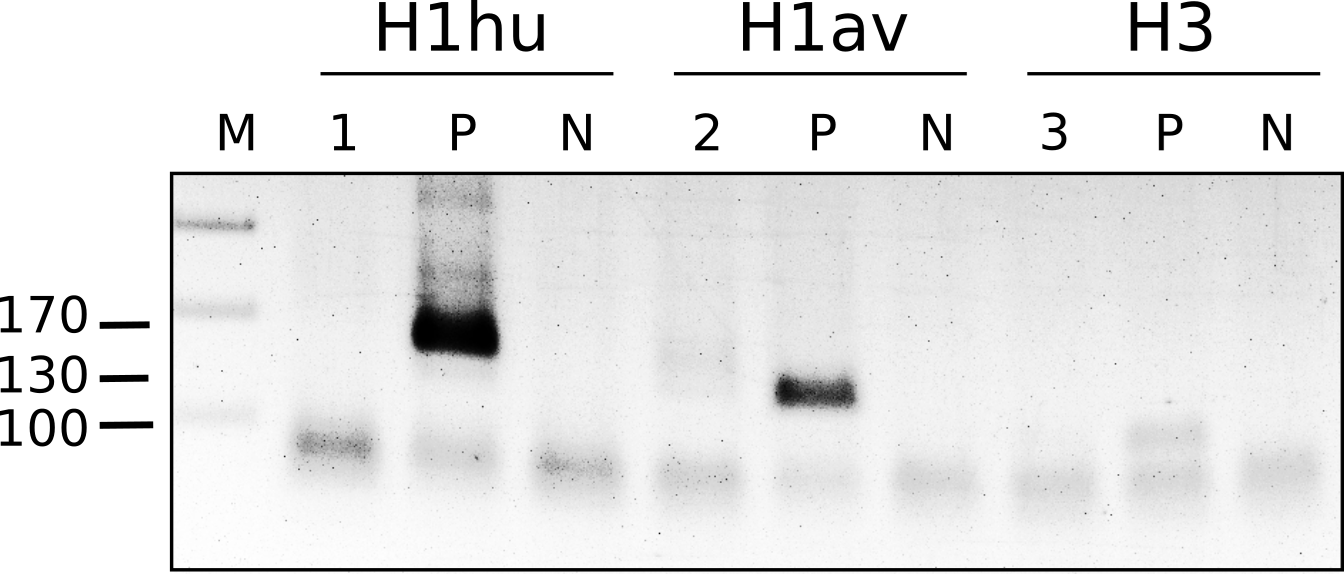

Supplement: Supplementary file 2 [file IRV-10-504-s002.tif]

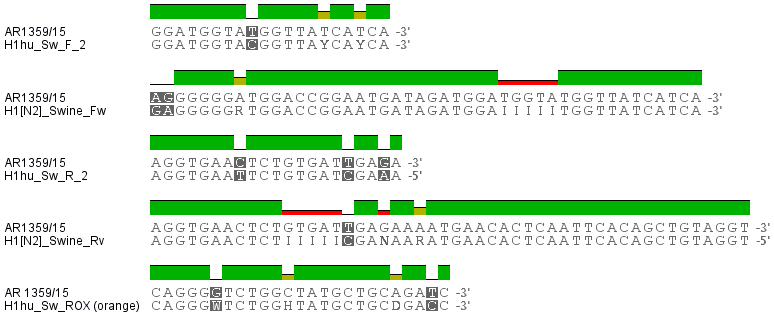

Supplement: Supplementary file 3 [file IRV-10-504-s003.tif]

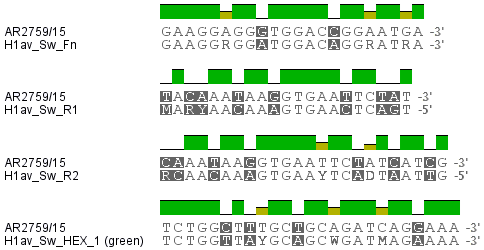

Supplement: Supplementary file 4 [file IRV-10-504-s004.tif]

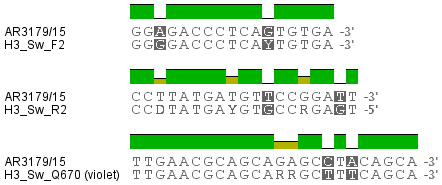

Supplement: Supplementary file 5 [file IRV-10-504-s005.tif]
